# Petitioning the FDA to Improve Pharmaceutical, Device and Public Health Safety by Ordinary Citizens: A Descriptive Analysis

**DOI:** 10.1371/journal.pone.0155259

**Published:** 2016-05-12

**Authors:** Brian K. Chen, Y. Tony Yang, Xi Cheng, John Bian, Charles L. Bennett

**Affiliations:** 1 Department of Health Services Policy and Management, Arnold School of Public Health, the University of South Carolina, Columbia, South Carolina, United States of America; 2 College of Health and Human Services, George Mason University, Fairfax, Virginia, United States of America; 3 South Carolina Center of Economic Excellence for Medication Safety and Efficacy, South Carolina College of Pharmacy, the University of South Carolina, Columbia, South Carolina, United States of America; Royal College of Surgeons, IRELAND

## Abstract

The United States Constitution protects the right of citizens to petition the government for “a redress of grievances.” This right has important implications for citizens desiring to advance the public health by petitioning administrative agencies, such as the Food and Drug Administration, to take safety actions. We examined a total of 1,915 petitions filed between 2001 and 2013 to investigate the outcomes of citizen petitions that address public health concerns. We found that most petitions were filed by manufacturers against other manufacturers. Only 346 (18%) of all petitions were submitted by individuals and non-profit organizations, and 178 (87.3%) of these petitions with a final response were denied. On average, these petitions required 2.85 years for a final agency decision, and many decisions remain pending 10–13 years after their initial submission. The great majority of the approved requests included some form of risk communication, such as labeling changes, boxed warnings or placement of a drug into a Risk Evaluation and Mitigation Strategy. As a policy instrument to improve the safety of medical and food products, the citizen petition process requires sophisticated legal and scientific expertise, and may not represent a viable route for ordinary citizens to petition the FDA to “redress grievances.”

## Introduction

Based on the First Amendment right of citizens to “petition the Government for a redress of grievances,” Title 21, Section 10.30 of the Code of Federal Regulations stipulates that citizens may request the Food and Drug Administration (FDA) to “issue, amend, or revoke a regulation or order or take or refrain from taking any other form of administrative action.”[1] These petitions have the potential to protect the public’s health. However, the citizen petition process has primarily been used by for-profit industries, often to deter competition. Historically, one fifth of these petitions have been successful [2]. Ordinary citizens or non-profit organizations can petition the FDA regarding safety issues related to drugs, devices or other items (generally food, tobacco, cosmetics or FDA regulations).

We reviewed the content and outcomes of petitions *not* filed by pharmaceutical or device manufacturers to provide empirical data on the types of individuals or organizations who submit citizen petitions, the nature of their petitions, and the historical likelihood of success for these petitions. We found that overall, only approximately 12.7% of petitions result in a favorable outcome, that the majority of petitions are denied because petitioners fail to present sufficient and/or convincing evidence, that the FDA sometimes denies petitions that are legally and scientifically sound due to unfavorable cost-benefit assessments, that the FDA prefers to grant only incremental requests rather than sweeping changes, and that organizations and professionals with legal and scientific expertise are more likely to receive a positive response from the FDA.

## Materials and Methods

We reviewed 1,915 citizen petitions filed with the FDA from 2001 to 2013. These petitions are publicly available at www.regulations.gov, and searchable using the term “FDA-YEAR-P-“, where YEAR is a four-digit numeral indicating the year in which a petition is filed. Petitions with a final FDA decision include a letter in which the agency details the nature of the petition requests and the reasoning for its decision. Overall, 18% of the 1,915 FDA citizen petitions were filed by individuals or organizations other than manufacturers. In this article, we focus on the 346 petitions filed by individuals and organizations (available in S1 File), after abstracting petitioner information from the 1,915 FDA citizen petitions.

Information on submission/decision dates, outcome, petitioner(s), and reason(s) for decisions was extracted from the FDA decision letters available in the docket folder for each decided citizen petition at the regulations.gov website. The abstraction was conducted by two attorneys and a research assistant. We separated outcomes into four categories–(a) petition granted in its entirely, if the FDA granted all of the requests contained in the petition; (b) petition granted partially with a substantive outcome, if the FDA granted at least one request leading to a decision that can potentially and directly improve public health; (c) petition granted partially with no substantive outcome, if the FDA granted at least one request that *in itself alone* is unlikely to lead to any changes in public health measures, and (d) petition denied in full.

The distinction between (b) and (c) is a subtle one, and may appear to require subjective evaluation. However, concrete examples may provide context for the distinction. If a petition requests multiple FDA actions, and the FDA grants only one requesting a labeling change for a particular drug, then we consider this result to be in category (c)–one with a substantive outcome. On the other hand, if a petition with multiple requests is granted only with respect to a request that the FDA forward information to another government agency or a third party or hold a meeting by a certain date, then we categorize this result as (d), partially granted with no substantive outcome. The key distinguishing feature that separates a “substantive” approval from a non-substantive one is that the former is in itself a safety action, such as a labeling change, placement on a safety advisory list, or marketing withdrawal, rather than preliminary steps such as information transmission to a different agency, establishment of a meeting date or permission to attend a meeting.

We separated the reasons for petition denial into four categories: (1) insufficient legal basis, (2) insufficient scientific or factual basis, (3) insufficient legal and scientific basis and (4) moot.

Because the Code of Federal Regulations set forth specific legal requirements and subject matter jurisdiction over citizen petitions, failure to meet these requirements will result in a denial on procedural grounds (category (1)). Category (2), “insufficient scientific/factual basis”, is a broad one that includes context-specific denials. Broadly speaking, however, common reasons for failure to persuade the FDA because of insufficient scientific or factual rationale include (2a) disagreement with the petitioner’s scientific basis for the requested action(s); (2b) lack of new information not previously considered by the FDA, or unacceptable forms of evidence such as personal communication or anecdotal evidence; (2c) belief that other preexisting solutions or framework sufficiently address petitioners’ concerns; (2d) lack of cost-benefit justification for the requested action(s) or (2e) mistaken fact. Petitions that are denied both on insufficient scientific or legal grounds are placed in category (3). Because of the time required for agency response, some requests are moot (category (4)) as a result of changes in the scientific or factual environment or following agency actions independent of the petitioner’s requests.

If abstractors disagree on the categorization of the outcome or reason for denial, the lead author determined the final categorization. The status of FDA decisions on the petitions is current as of March 28, 2016.

## Results

**Fig 1:**
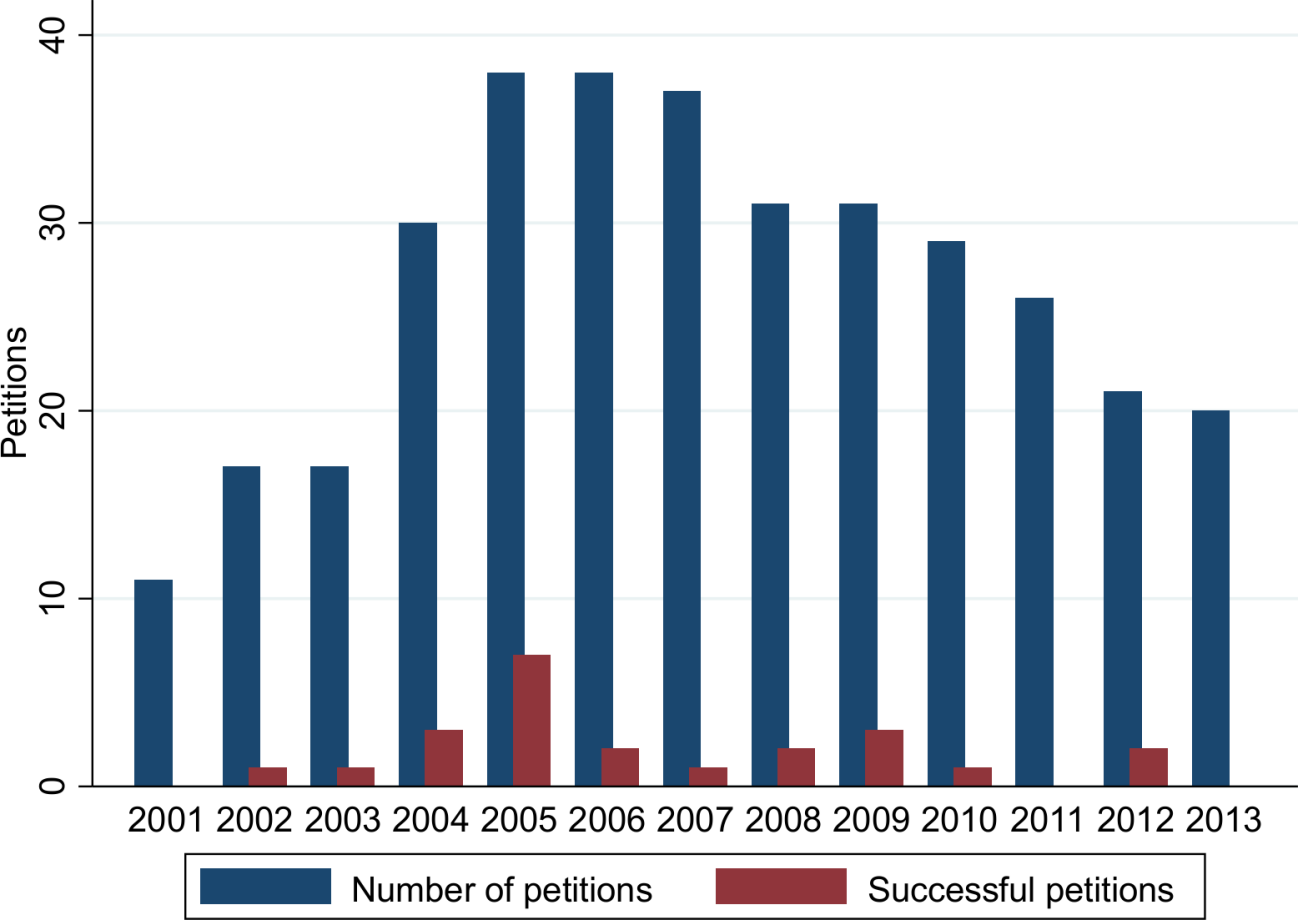
FDA Citizen Petitions over the Years. Successful petitions attributed to the year of filing, not to the year of decision. Source: www.regulations.gov.

**Fig 2:**
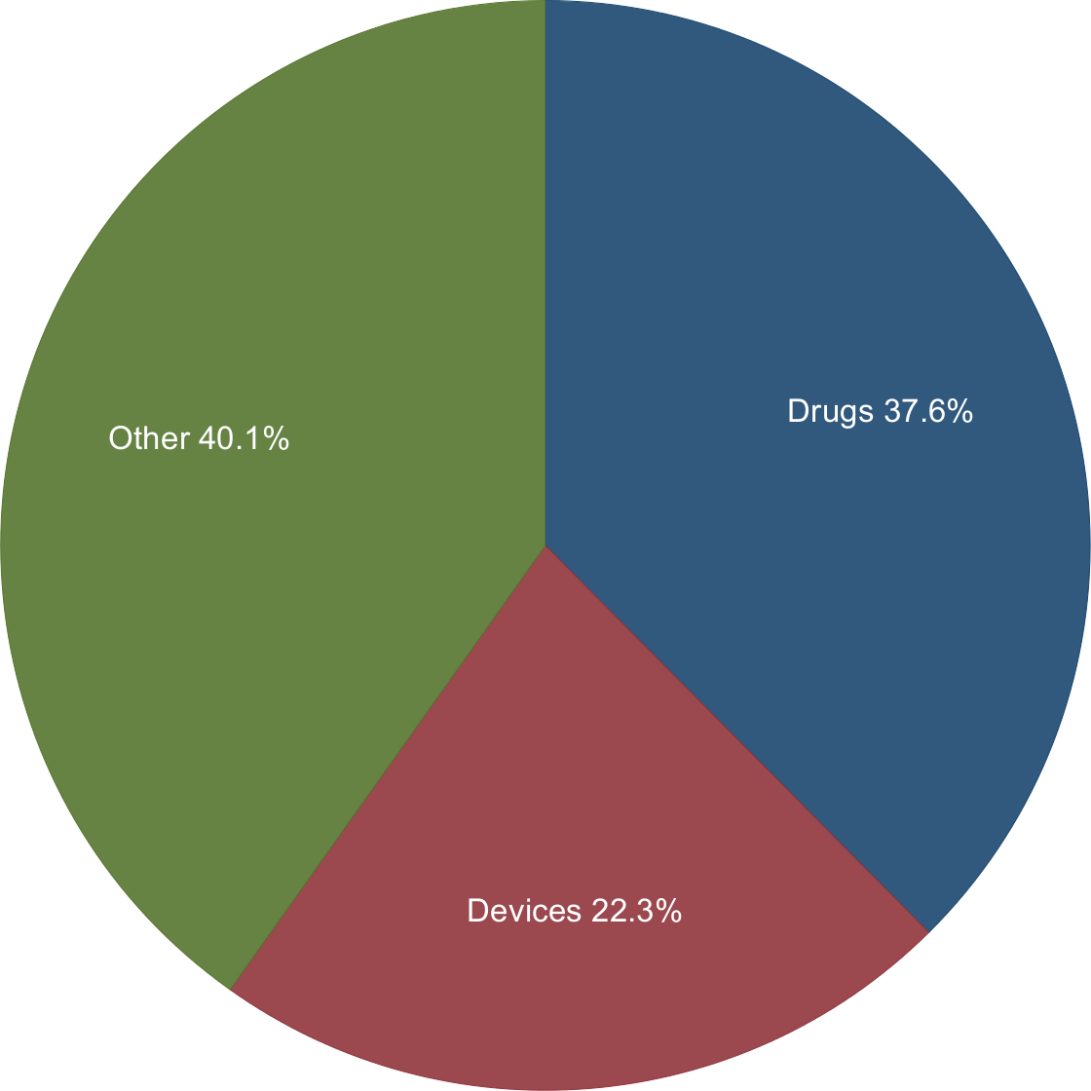
Subject Matter of Petitions. The category “other” includes petitions related to food, cosmetics, tobacco or regulations. Source: www.regulations.gov.

**Fig 3:**
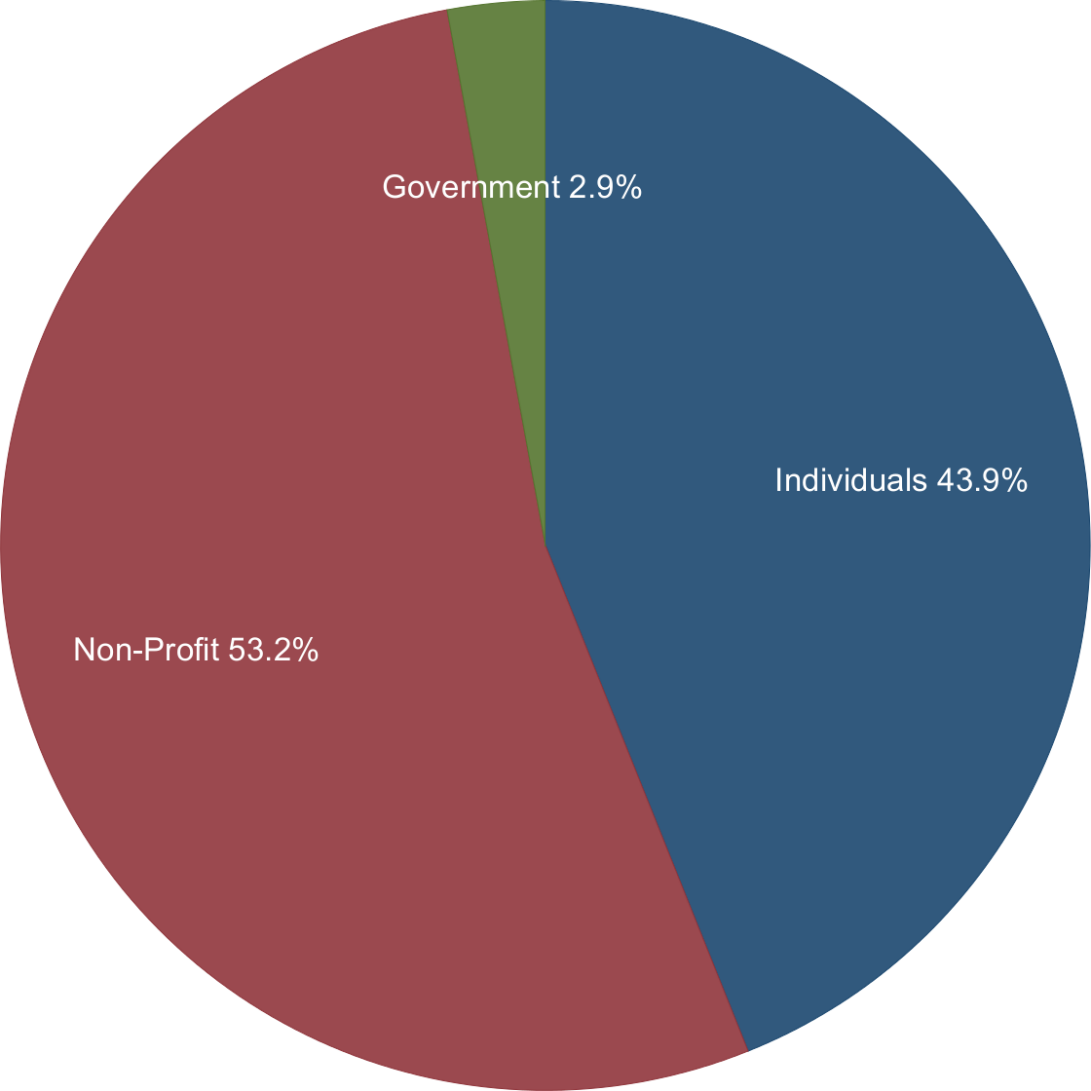
Types of Petitioners by Lead Petitioner. Source: www.regulations.gov.

**Fig 4:**
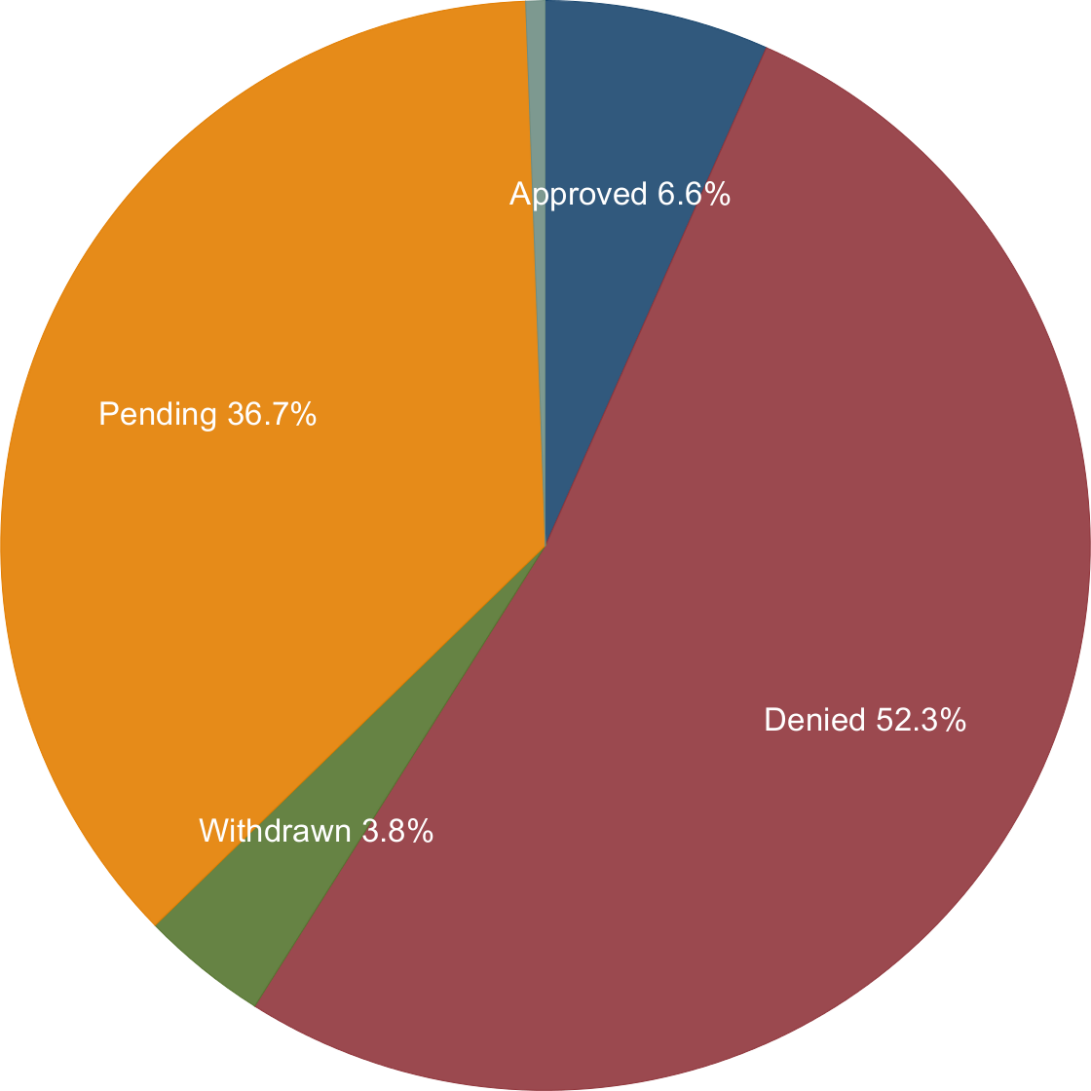
FDA Decisions on Petitions Filed between 2001 and 2013. Approved petitions include partially approved petitions with a substantive outcome. Denied petitions include petitions that are in essence denied. Source: www.regulations.gov.

Fig 1 shows the number of FDA citizen petitions from 2001–2013 and the number of successful petitions attributed to the year of filing (rather than the year of decision). Overall, 130, 77, and 139 citizen petitions were for drugs, medical devices and other subjects (generally food, cosmetics, tobacco or FDA regulations), respectively (Fig 2). Individuals filed 43.9% of these petitions (Fig 3). Five petitions (1.4%) were granted in their entirety, and 18 petitions (5.2%) were granted in part with a substantive outcome (See Table 1 for a description of the 23 petitions that were successful at least in part). Although 21 (6.1%) other petitions were partially granted, these were essentially denials as they approved non-substantive requests (Fig 4 and Table 2). Among 204 petitions with a final response (59% of all studied petitions), 23 (12.7%) had at least one substantive request granted. The time required to decision was a mean of 3.1 years for approval and 2.77 years for denial.

**Table 1:** Summary of Successful Petitions to the FDA (2001–2013).

Subject	Type	File Date	Dec. Date	Outcome
Permit health claims related to **nuts** and Coronary Heart Disease	Other	8/28/02	3/14/03	Partial Approval
Modify the test interaction section and labeling of **Premarin**	Drug	8/1/03	9/21/04	Partial Approval
Authorize a health claim for p-glucan soluble fiber from **barley products**	Other	8/3/04	5/22/06	Approval
Amend regulations on health claims for **Vitamin D**	Other	Not clear	9/29/08	Approval
**Partially hydrogenated oils** (PHOs) in human food	Other	11/29/09	6/16/15	Partial Approval
Additional warnings for **thalidomide**	Drug	Not clear	5/25/06	Partial Approval
Investigate **ibuprofen** manufacturers and require labeling changes	Drug	2/15/05	6/22/06	Partial Approval
Requests that FDA revise the labeling for **glycoprotein (GP) Ilb/IIIa inhibitors**	Drug	3/6/05	4/10/13	Partial Approval
Withdraw certain **Cox-2 Inhibitors**	Drug	1/24/05	7/26/05	Partial Approval
Allow claims that **whole oat** reduce the risk of CHD	Other	11/9/05	5/1/08	Approval
Recommend labeling changes and rescheduling of **tramadol** under the Controlled Substances Act (3 petitions)	Drug	10/25/05; 11/2/0511/15/05;	3/22/16	Partial approval
Require manufacturers of **fluoroquinolones** to take various safety actions	Drug	8/29/06	7/24/08	Partial Approval
Withdraw **oral sodium phosphate** (OSP) or add a black box warning about renal failure	Drug	9/20/07	12/11/08	Partial Approval
Require REMS, a Dear Doctor letter and black box warning for botulinum toxin	Drug	1/23/08	4/30/09	Partial Approval
Determine that **wheat gluten** as an excipient is not generally recognized as safe or to disclose its inclusion in drugs	Drug	6/2/08	5/12/15	Partial Approval
Allow access to **promising HCV investigational drugs**	Drug	9/29/09	4/23/10	Partial Approval
Immediately ban the weight loss drug **Meridia (sibutramine)**	Drug	12/3/09	1/3/11	Approval
Labeling changes and safety warnings for **epoetin alfa** and **darbepoetin alfa**	Drug	9/1/09	6/24/11	Partial Approval
**Gadolinium-based contrast agents** relating to the risk of nephrogenic systemic fibrosis	Drug	4/1/2010	12/20/10	Partial Approval
Compliance of **Form FDA 3429 General Device Classification questionnaire**	Other	7/10/12	3/4/13	Approval
Change **opioid analgesic labels**	Drug	7/25/12	9/10/13	Partial Approval

The category “other” includes food, cosmetics, regulations, or tobacco.

**Table 2:** FDA Decisions by the Numbers.

Decision type	Number	% of Total
Answered a clarification question	2	0.6%
Approved	5	1.4%
Approved in part (with substantive outcome)	18	5.2%
Denied	160	46.2%
Essentially denied (approved in part with no substantive outcome)	21	6.1%
Withdrawn	13	3.8%
Pending	127	36.7%
Grand total	346	100.0%

**Fig 5:**
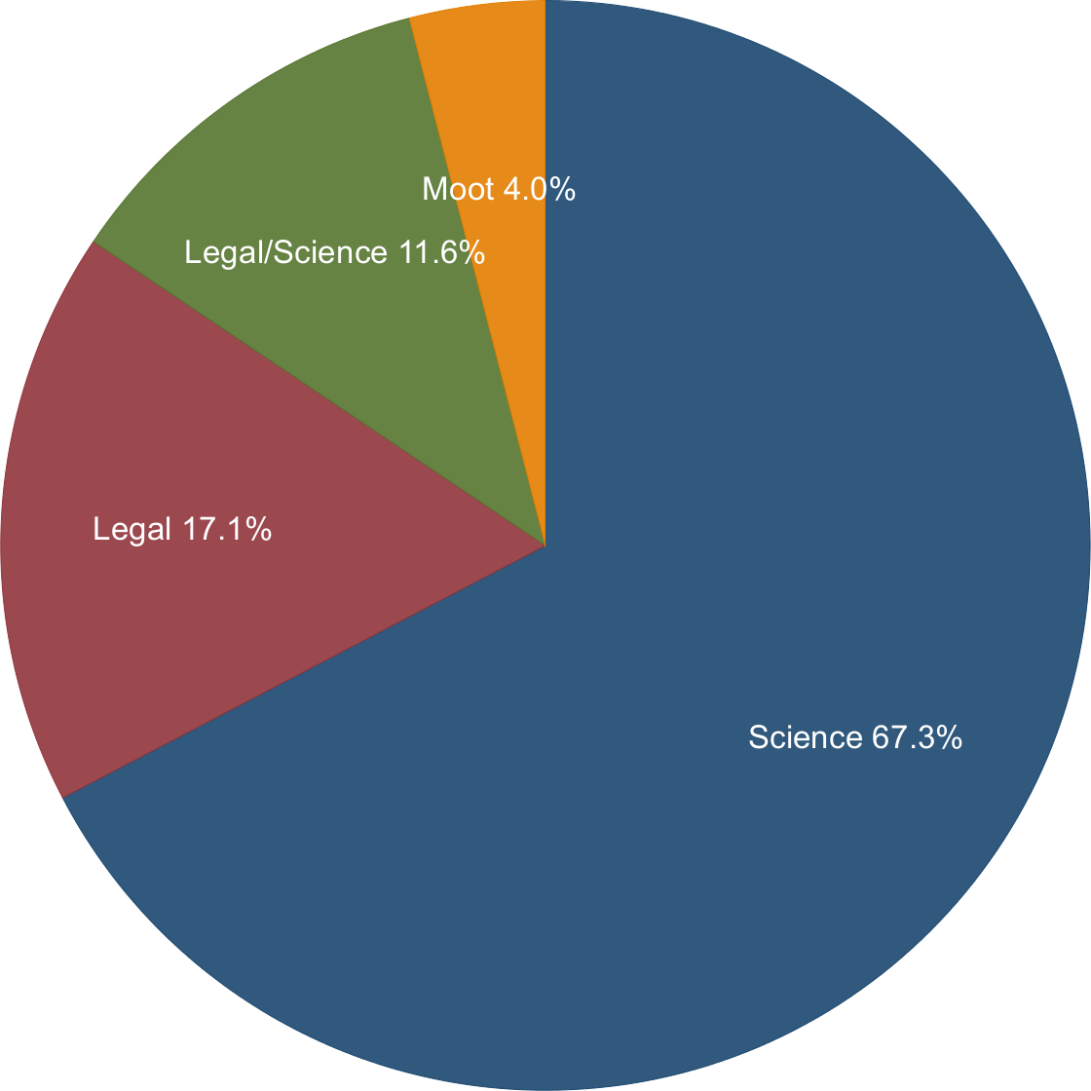
Reasons for Petition Denials. Denied petitions include petitions that are denied in part. Source: www.regulations.gov.

Of 199 citizen petitions with a denial, 67.3% (134) were denied on scientific grounds, 17.1% (34) were denied on legal grounds, 11.6% (23) were denied for both legal and scientific reasons, and 4% (8) were denied as moot (Fig 5 and Table 3). Below we highlight examples of petitions reviewed by the FDA.

**Table 3:** Reasons for Denials by the Numbers.

Reason for Decision	Number	% of Total
Of 199 petitions with a final negative decision		
Insufficient scientific or factual grounds	134	67.3%
Moot	8	4.0%
Insufficient legal basis	34	17.1%
Insufficient scientific and legal basis	23	11.6%
Grand total	199	100.0%

### Petitions Granted in Full

Of petitions with a response from the FDA between 2001 and 2013, only two non-health claim public interest petitions were granted in their entirety. One such petition successfully questioned the validity of an FDA questionnaire. Only a single petition granted in full persuaded the FDA to take substantial safety action with respect to a prescription drug. FDA-2009-P-0595, filed by the non-profit consumer rights advocacy group Public Citizen, requested that the FDA immediately ban the weight loss drug sibutramine based on early results from the Sibutramine Cardiovascular Outcomes Trial (SCOUT trial) [3] and safety events reported to FDA's Adverse Event Reporting System (FAERS).

Because of the large numbers of petitions that are granted in part and denied in part, or denied in their entirety, below we only highlight illustrative examples of petitions falling in these categories.

### Petitions Granted in Part and Denied in Part, but with a Favorable Substantive Outcome

Other petitions were granted in part and denied in part, when they included multiple requests. Almost of all these granted requests asked for labeling changes, boxed warnings, or other types of safety communications. In FDA-2012-P-0818, FDA agreed that changes to the labeling of extended-release and long-acting opioid analgesics were needed to more effectively communicate to prescribers the serious risks associated with these drugs. FDA- 2010-P-0179 approved labeling changes for gadolinium-based contrasting agents that reclassified gadolinium-associated nephrogenic systemic fibrosis syndrome as being caused by three specific agents, whereas previously the warnings had been for the entire class of these agents. In FDA-2009-P-0426, a boxed warning was approved for Epogen/Procrit to state that a maintenance target hemoglobin/hematocrit range had not been established for chronic kidney disease patients. The FDA agreed that a Risk Evaluation and Mitigation Strategy (REMS) and additional warnings would be necessary for botulinum toxin products for the potential distant spread of toxin effects (FDA-2008-P-0061).

### Petitions Granted in Part and Denied in Part, but with No Substantive Outcome

While some petitions were in theory granted in part, we consider them in essence denied because no substantive requests were granted. Examples include requests to convene a meeting or forward information to another department within the FDA, with no guarantee or promise of any other non-procedural action. FDA-2013-P-0735, for example, granted a request to forward petitioner’s comment to the FDA Executive Secretariat; and FDA-2012-P-0857 approved a request to *ask* Johnson & Johnson to submit “all internal documents … from the Risperdal litigation.”

### Petition Denials

#### Denials due to lack of or disagreement with petitioners’ statement of grounds

Most petitions to the FDA were denied. We summarize in Table 4 the most common categories of reasoning given for the denial. The vast majority of denials resulted from FDA’s disagreement with the petitioner’s scientific rationale for requested actions. These denials are akin to the petitioners failing to carry their “burden of persuasion,” or the duty to establish his or her right to administrative relief by convincing the FDA that the facts asserted are true and support the requested actions. For these denials, the FDA provided detailed, point-by-point rebuttals to the petitioner’s scientific basis for the requested actions. These denials are often highly specific to the petitions. At times, however, petitioners failed to fulfill their “burden of production” by not providing the minimal amount of justification required. Examples include FDA-2006-P-0347 (denying a petition based on individual case reports, personal experience, and third party testimonials), FDA-2006-P-0389 (a petition based on statements from an unnamed speaker), and FDA-2005-P-0121 (petitioners have not presented the sort of analysis of primary data and overall analysis of the studies that would be needed to support a new claim).

**Table 4:** Common subcategories for petition denials on scientific and legal grounds.

Reason	Explanation	Frequency	% of Total
Scientific Rationale			
Disagreed with petitioner's interpretation	Although petitioner met the burden of production (in providing evidence), petitioner failed to carry the "burden of persuasion" because the FDA disagreed with petitioner's conclusion and/or need for the requested action	69	43.9%
Failure to produce sufficient data	Petitioner failed to meet the burden of production of evidence, failing to produce sufficient evidence for the FDA to make a decision	65	41.4%
Preexisting solution	The FDA believed that a preexisting framework or solution rendered the requested action unnecessary.	13	8.3%
Risk-benefit considerations	The FDA, although agreeing with petitioner's analysis and data, nevertheless denied the requested action because it deemed that the benefits do not justify the cost or are limited in comparison with the risks.	8	5.1%
Mistake	Petitioner made a mistake of fact that rendered the request unactionable. An example is requesting "reinstatement" of a withdrawn product that was never approved to begin with.	2	1.3%
		157*	100.0%
Legal Rationale			
No legal grounds for request	Petition did not follow the legal requirements for a citizen petition as set forth in CFR Title 21, Section 10.30 or other applicable laws and regulations.	21	61.8%
No jurisdiction	Subject of the petition did not fall under the jurisdiction of the FDA.	13	38.2%
		34	100.0%

*23 petitions denied on both legal and scientific grounds are analyzed primarily for the scientific rationale for denial in this table, and 8 petitions denied as moot are not included in Table 4.

#### Denials due to unfavorable risk/benefit tradeoffs

Even when FDA appeared to accept the scientific basis for potential harm of certain drugs or devices, FDA sometimes took a risk-benefit approach to sanction current practices or guidelines. In FDA-2009-P-0362, FDA declined to issue an order for inspection of *every* stent, heart valve, and filter because doing so would be cost-prohibitive. Other denials using such risk-benefit approaches include FDA-2003-P-0336 (low risk of nefazodone hepatoxicity is justified by its potential benefit), FDA-2006-P-0542 (pill counting by weight rather than number is justified), FDA-2005-P-0192 (risks of PDE5 inhibitors may not be sufficiently serious relative to their benefits), and FDA-2006-P-0453 (general reformulation requirement for all addictive drugs is not justified).

#### Denials due to legal procedural reasons

Approximately 17% of citizen petitions were denied on legal or procedural grounds, sometimes without addressing the merits. A common reason for denial is requesting an action for enforcement, which is not appropriate through the citizen petition process. Examples include FDA-2011-P-0777 (“To the extent that your citizen petition requests that FDA initiate enforcement action(s), please note that this type of request is not within the scope of FDA's citizen petition procedures”). Other requests not within the scope of or not following procedures required for the citizen petition process were also denied. For example, FDA-2009-P-0111 and FDA-2009-P-0186 were denied because the requests were deemed to be subject to procedures set forth in specific regulations, and the FDA felt that the submission did not comply with these procedures.

Petitions have also been denied because requested courses of action are not within FDA’s jurisdiction. These include FDA-2007-P-0346 (the Environmental Protection Agency, not FDA, has regulatory authority over public water supplies, so a “petition requesting that FDA ban the use of synthetic fluoride compounds in public drinking water is denied”), and FDA-2003-P-0014/0291 (Petitioner's concern over mail order/internet company sales of contact lenses without a valid current prescription is better addressed to the Federal Trade Commission (FTC), as it is an authority granted to the FTC by the Fairness to Contact Lens Consumers Act (FCLCA, 15 USC 7601–7610)).

### Higher Success Rates with Professionals

Seventeen of the 23 successful petitions (73.9%) were submitted by organizations or physicians. Public Citizen submitted four successful petitions. The Attorneys General of Connecticut and Illinois each submitted petitions that were accepted in part [4, 5]. These petitions requested in part that the FDA require the manufacturer to add “Black Box” warnings describing severe adverse events (thalidomide-induced venous thromboembolism and quinolone-induced tendon rupture, respectively). The petitions included data prepared by an academic pharmacovigilance group or Public Citizen, respectively [4–6]. These were the first two successful citizen petitions ever filed by state Attorneys General. Four petitions previously filed by these Attorneys General did not include empirical data and were denied [6].

## Discussion

Between 2001 and 2013, twenty-three petitions filed with the FDA by citizens have been successful in part or in total. The FDA denied 87.3% of petitions by individuals, non-for-profit organizations and advocacy groups and other governmental agencies. Common grounds for denials of these petitions include: request for actions not under FDA jurisdiction; failure to provide new information not previously considered by the FDA; and failure to present an overall analysis of a factual basis to support the petition. In many cases, the FDA disputes the petitioners’ interpretation of supporting data. Even when the FDA agreed with the petitioners’ data analysis, the FDA has denied requests based on assessments of cost versus benefits of requested actions.

Second, the time to a decision is potentially very long, with some petitions still pending 10 to 13 years after submission. Among decisions that received a final response, one petition took almost 13 years for resolution (FDA-2001-P-0283). Possible reasons for the long delay include agency resource constraints to address often highly technical petitions, and the lack of legal requirement for a final response within a given time period except for citizen petitions related to an Abbreviated New Drug Application, which must be responded to within 90 days of petition receipt [1]. Overall, the mean time to FDA issuance of decision was 2.85 years. A number of petitions were denied, or rendered moot, because market conditions and/or the weight of the evidence had changed so much by the time the FDA made a final decision.

Third, the scope of FDA citizen petitions is limited to rules and regulations over which the Commissioner has authority and jurisdiction. As a result, not all actions necessary to improve the safety of prescription drugs, medical devices, and food and herbal supplements can be requested. In our analytical sample, for example, FDA denied several petitions and designated the Federal Trade Commission or the Environmental Protection Agency as the competent authority. Most importantly, however, FDA appears to be reluctant to make sweeping changes, preferring to take incremental steps, approving mostly only risk communications.

## Conclusion

We conclude that thus far, citizen petitions filed by “ordinary” citizens are rarely successful. Future petitioners should consider several strategies in submitting citizen petitions. First, petitioners must comply with the legal requirements of CFR Title 21, Section 10.30 (which include (a) actions requested, (b) statement of grounds, (c) environmental impact, which is generally required for petitions related to food or color additives, drugs, biological products, animal drugs or certain medical devices (see Title 21, Part 25 of the Code of Federal Regulations for further detail), (d) economic impact (if requested by the FDA), and (e) certification/signature). In particular, petitioners should ascertain that the actions requested are under the authority and jurisdiction of the FDA Commissioner. The vast majority of citizen petitions are denied because they fail either to meet the burden of production or persuasion by not providing sufficient data for the FDA to make a determination, or by not providing sufficiently persuasive evidence to convince the FDA to take or refrain from taking an action. Such evidence will likely derive from an extensive, consistent body of peer-reviewed literature or randomized controlled trials that strongly support the appropriateness of and need for the petitioner’s request(s). In requesting an FDA action, it is also important not to overreach, and to tailor the request to the identified need as narrowly as possible. Giving the technical expertise required for a successful petition, partnering with an organization with regulatory expertise and pharmacovigilance programs (as occurred with thalidomide and fluoroquinolones) in filing citizen petitions should be considered.

Many citizen petitions remain pending, including petitions that address important public health concerns related to menthol in tobacco, antibiotic use in food-producing animals, the naming of high fructose corn syrup, and a number of important classes of commonly prescribed drugs. Two such citizen petitions focusing on additional fluoroquinolone toxicities were submitted in 2014 [7, 8]. An FDA advisory panel recently voted overwhelmingly to recommend labeling changes for fluoroquinolones in view of these severe adverse events [9]. FDA responses are pending. It is hoped that, building on the Attorneys General’s experiences, this partnership of scientific and legal expertise may prove successful.

## Supporting Information

S1 File Citizen Petition Data. Listing of all citizen petitions filed in the public interest by petitioners other than pharmaceutical or device manufacturers, 2001–2013.
(XLSX)
